# Requirements for human embryonic stem cells

**DOI:** 10.1111/cpr.12925

**Published:** 2020-10-19

**Authors:** Jie Hao, Jiani Cao, Lei Wang, Aijin Ma, Si Chen, Jinfeng Ding, Liu Wang, Boqiang Fu, Yu Zhang, Xuetao Pei, Peng Xiang, Qiyuan Li, Yong Zhang, Jiaxi Zhou, Shijun Hu, Junying Yu, Jun Wei, Huanxin Zhu, Glyn Stacey, Tongbiao Zhao, Qi Zhou

**Affiliations:** ^1^ State Key Laboratory of Stem Cell and Reproductive Biology Institute for Stem Cell and Regeneration Institute of Zoology National Stem Cell Resource Center Chinese Academy of Sciences Beijing China; ^2^ Chinese Society for Stem Cell Research Shanghai China; ^3^ China National Institute of Standardization Beijing China; ^4^ China National Institute of Metrology Beijing China

**Keywords:** human embryonic stem cells, quality control, standard

## Abstract

‘Requirements for Human Embryonic Stem Cells’ is the first set of guidelines on human embryonic stem cells in China, jointly drafted and agreed upon by experts from the Chinese Society for Stem Cell Research. This standard specifies the technical requirements, test methods, test regulations, instructions for use, labelling requirements, packaging requirements, storage requirements and transportation requirements for human embryonic stem cells, which is applicable to the quality control for human embryonic stem cells. It was originally released by the China Society for Cell Biology on 26 February 2019 and was further revised on 30 April 2020. We hope that publication of these guidelines will promote institutional establishment, acceptance and execution of proper protocols, and accelerate the international standardization of human embryonic stem cells for applications.

## 1 SCOPE

This document specifies the technical requirements, test methods, test regulations, instructions for use, labelling requirements, packaging requirements, storage requirements and transportation requirements for human embryonic stem cells.

This standard is applicable for the quality control of human embryonic stem cells.

## 2 NORMATIVE REFERENCES

The following content constitutes indispensable articles of this standard through normative reference. For dated references, only the edition cited applies. For undated references, only the latest edition (including all amendments) applies.

WS213 *Diagnosis for hepatitis C*

WS273 *Diagnosis for syphilis*

WS293 *Diagnosis for HIV/AIDS*

T/CSCB 0001‐2020 *General requirements for stem cells*

*Pharmacopoeia of the People's Republic of China, Volume Ш*

*National Guide to Clinical Laboratory Procedures*

*Ethical guidelines for research of human embryonic stem cells*

## 3 TERMS, DEFINITIONS AND ABBREVIATIONS

### 3.1 Terms and definitions

For the purposes of this document, the terms and definitions in T/CSCB 0001‐2020 and the following terms and definitions apply.

#### 3.1.1 Human embryonic stem cell

The undifferentiated cells derived from pre‐implantation human embryo, which are able to self‐renew indefinitely in vitro and can differentiate into all cells of the three embryonic germ layers.

#### 3.1.2 Karyotype

Chromosomal characteristics of a cell in the mitotic metaphase, including chromosome number, length, centromere position, satellites, primary constriction and secondary constriction.

#### 3.1.3 Teratoma

A type of benign tumour containing representative differentiated tissues and cells from all three embryonic germ layers.

#### 3.1.4 Mycoplasma

A class of small and facultatively anaerobic prokaryotic microbes which lack cell walls and can survive independently.

### 3.2 Abbreviations

The following abbreviations are applicable for this document.

DNA: deoxyribonucleic acid.

EBV: Epstein‐Barr virus.

HBV: hepatitis B virus.

HCMV: human cytomegalovirus.

HCV: hepatitis C virus.

HIV: human immunodeficiency virus.

HTLV: human T‐lymphotropic virus.

PCR: polymerase chain reaction.

STR: short tandem repeat.

TP: Treponema pallidum

## 4 TECHNICAL REQUIREMENTS

### 4.1 Source materials and auxiliary materials

1. For the harvesting of human biological source material (source materials), the *Ethical guidelines for research on human embryonic stem cells* shall be followed.
2. The source materials, reagents, consumables and other auxiliary materials and/or supplies (eg gases) shall meet the requirements of T/CSCB 0001‐2020.
3. The donor shall be negative for HIV, HBV, HCV, HTLV and TP.

### 4.2 Primary quality attributes

Primary quality attributes shall meet the requirements in Table 1.

**Table 1:** Primary quality attributes

Attributes	Requirements
Cell morphology	Cells grown in 2D conditions shall exhibit growth as colonies with clear boundaries, high nuclear‐cytoplasmic ratios and uniform morphology. Within each colony, cell‐cell contact should be tight.
Cell authentication	Cell lines shall have a known unique genetic profile to facilitate exclusion of cross‐contamination with other cells and confirm donor origin. Human short tandem repeat (STR) analysis shall include the following 16 human‐specific alleles: D3S1358, vWA, FGA, D8S1179, D21S11, D18S51, D5S818, D13S317, D16S539, TH01, TPOX, CSF1PO, D7S820, AMEL, PentaE and PentaD.
Chromosome karyotype	46, XX or 46, XY
Cell viability	≥90% before cryopreservation and ≥60% after resuscitation
Cell markers	Cell surface markers: ≥70.0% of the cell population express any two of the following genes: SSEA3, SSEA4, TRA‐1‐60, TRA‐1‐81, for example, TRA‐1‐81–positive rate ≥70.0% and SSEA4–positive rate ≥70.0%; intracellular markers: OCT4‐positive rate ≥70.0% and NANOG‐positive rate ≥70.0%
Teratoma formation	Shall generate teratomas with cells from all three germ layers
Microorganisms	Shall be negative for fungi, bacteria, mycoplasma, HIV, HBV, HCV, HTLV, EBV, HCMV and TP.

### 4.3 Process control

The process of cell expansion, cryopreservation and resuscitation shall follow the requirements of T/CSCB 0001‐2020.

## 5 INSPECTION METHODS

### 5.1 Cell morphology

Observe the morphology of cells grown in 2D condition using a microscope.

### 5.2 Cell authentication

The method in Appendix A shall be followed.

### 5.3 Chromosome karyotype

The method in the *Pharmacopoeia of the People's Republic of China* (Volume III) shall be followed.

### 5.4 Cell viability

The method in Appendix B shall be followed.

### 5.5 Cell markers

The method in Appendix C shall be followed.

### 5.6 Teratoma formation

The method in Appendix D shall be followed.

### 5.7 Microorganisms

#### 5.7.1 Fungi

The method in *Pharmacopoeia of the People's Republic of China* (Volume III) shall be followed.

#### 5.7.2 Bacteria

The method in *Pharmacopoeia of the People's Republic of China* (Volume III) shall be followed.

#### 5.7.3 Mycoplasma

The method in *Pharmacopoeia of the People's Republic of China* (Volume III) shall be followed.

#### 5.7.4 Human immunodeficiency virus

The method in WS 293 shall be followed.

#### 5.7.5 Hepatitis B virus

The method in the *National Guide to Clinical Laboratory Procedures* shall be followed.

#### 5.7.6 Epstein‐Barr virus

The method in the *National Guide to Clinical Laboratory Procedures* shall be followed.

#### 5.7.7 Treponema pallidum

The method in WS 293 shall be followed.

## 6 INSPECTION RULES

### 6.1 Sampling method

1. Cells produced from the same production cycle, same production line, same source, same passage and same method are considered to be the same batch.
2. Three smallest units of packaging shall be randomly sampled from the same batch.

### 6.2 Quality inspection and release

1. Each batch of products shall be subject to the qualify inspection before release, and inspection reports shall be attached.
2. The quality inspection items shall include all the attributes specified in 4.2.

### 6.3 Review inspection

Review inspection shall be performed by professional cytological testing institutions/laboratories as necessary.

### 6.4 Decision rules

1. Products that pass all requirements in 4.2 for the quality inspection for release are considered to be qualified. Products that fail to pass one or more requirements in 4.2 for the quality inspection for release are considered to be unqualified.
2. Products that pass all requirements in 4.2 for the quality review inspection are considered to be qualified. Products that fail to pass one or more requirements in 4.2 for the review inspection are considered to be unqualified.

## 7 INSTRUCTIONS FOR USAGE

The instructions for usage shall include, but not limited to:

1. Product name;
2. Passage number;
3. Cell numbers;
4. Production date;
5. Lot number;
6. Production organization;
7. Storage conditions;
8. Shipping conditions;
9. Contact information;
10. Operation manual ;
11. Execution standard number;

Note: according to what standards are the cells produced

- Manufacturing address;

Note: alternatively refers to the derivation laboratory

- Postal code;
- Matters that need attention.

## 8 LABELS

The label shall include but not limited to:

1. Product name;
2. Passage number;
3. Cell number;
4. Lot number;
5. Production organization;
6. Production date.

## 9 PACKAGE, STORAGE AND TRANSPORTATION

### 9.1 Package

The appropriate materials and containers shall be selected to ensure maintenance of the primary quality attributes of human embryonic stem cells.

### 9.2 Storage

1. T/CSCB 0001‐2020 shall be followed.
2. Productions should be stored in liquid nitrogen.

### 9.3 Transportation

1. T/CSCB 0001‐2020 shall be followed.
2. Cryopreserved cell products shall be transported in dry ice or liquid nitrogen.

## CONFLICT OF INTEREST

No potential conflicts of interest are disclosed.

## AUTHOR CONTRIBUTIONS

QZ and TZ contributed to conception and design. JH, JC, LW, AM and SC drafted and revised the manuscript. JD, LW, BF, YZ, XP, PX, QL, YZ, JZ, SH, JY, TW, HZ and GS critically read and revised the manuscript.

## Appendix A

### Cell authentication (pcr‐capillary electrophoresis)

**A.1 Instruments and equipment**

A.1.1 PCR‐Cycler.
A.1.2 Bench‐top centrifuge.
A.1.3 Genetic sequence analyzer.
A.1.4 Nucleic acid quantification machine.
A.1.5 Genetic map analysis software

**A.2 Reagents**

Short tandem repeat (STR) typing assay kit

**A.3 Testing protocol**

A.3.1 Extraction of cellular DNA

Perform DNA extraction according to the manufacturer's instructions.

A.3.2 Amplification of STR loci using fluorescent primers

Perform amplification according to the manufacturer's instructions.

A.3.3 Capillary electrophoresis of PCR products

Capillary electrophoresis testing and analysis shall be performed according to the manufacturer's instructions for the genetic sequence analyzer.

**A.4 Result analysis**

Alignment of the obtained STR typing map with the human standard STR map for comparison.

## Appendix B

### Cell viability test (Cell enumeration method)

**B.1 Instruments and equipment**

B.1.1 Microscope.
B.1.2 Haemocytometer.

**B.2 Reagents**

Unless otherwise stated, all reagents used shall be analytical grade. The water used for testing shall be deionized water.

B.2.1 Phosphate buffered saline (PBS): pH 7.4.
B.2.2 Trypan blue solution.

**B.3 Testing protocol**

B.3.1 Preparation of cell suspension

Harvest and suspend the cells with appropriate volume of DPBS. The cells in the haemocytometer shall be 20‐50 cells/mm^2^. Serial dilution is necessary if the number of cells exceeds 200 per haemocytometer.

B.3.2 Trypan blue staining

Evenly mix the Trypan blue solution (B.2.2) with the cell suspension (B.3.1) at a volume ratio of 1:1.

B.3.3 Cell counting

Load the haemocytometer with 10 μL of the trypan blue‐labelled sample (B.3.2). Make sure the entire chamber is filled with the testing sample. Stand for 30 seconds, count the stained cells and the total number of cells respectively.

For the 16 × 25 counting chamber, use the four 1 mm^2^ medium squares at the top left, top right, bottom left, and bottom right of the chamber (ie, 100 small squares) for counting.

For the 25 × 16 counting chamber, use the five 1 mm^2^ medium squares at the top left, top right, bottom left, bottom right, and centre of the chamber (ie, 80 small squares) d for counting.

When there are cells on the lines of the large square, only cells on the top line and left line of the large square can be counted (or alternatively only cells on the bottom line and right line).

**B.4 Calculation and analysis**

Cell viability is calculated according to equation (1):

(1) $$S=\left(M‐D\right)/M\times 100\%.$$

In the equation:

*S*——*viability of cells*

*M——total number of cells*

*D——number of stained cells*

The viability of cells is the mean of two duplicate samples. Two independent cell viability tests shall be performed on the same sample. The mean value of two independent viability tests is recorded as the viability of cells.

**B.5 Accuracy**

The absolute difference value between the two independent tests, under the same conditions, shall not exceed 10% of their arithmetic mean.

## Appendix C

### Detection of cell markers (flow cytometry)

**C.1 Instruments and equipment**

C.1.1 Flow cytometer.
C.1.2 Bench‐top centrifuge.
C.1.3 Ice‐making machine.
C.1.4 Electronic balance.

**C.2 Reagents**

Unless otherwise stated, all the reagents used shall be analytical grade. The water used in the experiment shall be Grade 1 water as stipulated in GB/T 6682.

C.2.1 Phosphate buffered saline (PBS): pH7.4.
C.2.2 Paraformaldehyde (PFA): Purity 95%.
C.2.3 Bovine serum albumin (BSA): Purity ≥98%.
C.2.4 Triton X‐100.
C.2.5 Antibodies.
C.2.6 Prepare the following solutions according to the relative requirements for flow cytometry: wash solution, fixing solution, blocking/permeabilization solution, antibody dilution solution.

**C.3 Sample storage**

The wash solution and fixed samples shall be stored at 2‐8°C. The fixing solutions shall be aliquoted, sealed, labelled, and stored at below –20°C. Antibodies shall be stored according to the manufacturer's instructions.

**C.4 Testing protocol**

C.4.1 Sample preparation and fixation

Collect samples by centrifuging single cell suspensions at 250 *g* for 3 minutes. Discard the supernatant. Resuspend the cells in an appropriate volume of fixing solution and incubate for 10 minutes in an ice bath. Wash the cell samples with an appropriate volume of wash solution for 3‐5 times (3‐5 minutes each time).

C.4.2 Blocking and permeabilization

Resuspend the fixed sample (C4.1) with the blocking/permeabilization solution and aliquot the cells into two independent samples, which will be used as a testing sample and an isotype control sample respectively. Incubate on ice for 20 minutes, then wash the samples with the wash solution.

C.4.3 Antibody incubation

Incubate the samples with the diluted antibodies or corresponding isotype controls according to the manufacturer's instructions.

C.4.4 Filtering and loading

Resuspend the samples with wash solution and then transfer the cell suspension into flow cytometry tube by filtering the samples through a mesh with 40 μm pores. Load the samples into the flow cytometer and perform testing according to the manufacturer's instruction.

C.4.5 Gating

Gate the target population of cells based on particle size and transparency, excluding cell debris and other irrelevant particles. The gating of positive staining cells shall be determined by the fluorescence intensity using isotype controls as a reference. Both positive and negative experimental controls shall be set up for gating and the following analysis.

**C.5 Analysis of results**

Analyze the results using software according to manufacturer's instructions.

## Appendix D

### Teratoma formation

**D.1 Instruments and equipment**

D.1.1 Haemocytometer.
D.1.2 Microscope.
D.1.3 Bench‐top centrifuge.
D.1.4 Embedding machine.
D.1.5 Microtome.
D.1.6 Tissue flotation bath.

**D.2 Reagents**

Unless otherwise stated, all the reagents used shall be analytical grade. The water used in the experiment shall be Grade 1 water as stipulated in GB/T 6682.

D.2.1 Phosphate buffered saline (PBS): pH 7.4.
D.2.2 Paraffin (melting point: 60°C).

**D.3 Testing protocol**

D.3.1 Preparation of cell samples
D.3.1.1 Cell digestion

Collect the cells by centrifuging at 250 *g* for 3 minutes and resuspending the cells gently in PBS, avoiding generation of air bubbles.

D.3.1.2 Cell counting

Calculate the concentration of living cells in suspension according to the method in Appendix B.

D.3.2 Cell transplantation

Inject 1 × 10^6^‐1 × 10^7^ human embryonic stem cells into 6‐8 weeks old immunodeficient mice subcutaneously, intramuscularly, or into the space between the seminiferous tubules below the tunica albuginea with a syringe.

D.3.3 Teratoma sampling and processing

Six‐10 weeks after transplantation with human embryonic stem cells (avoid allowing teratomas to grow beyond 15% of the mouse body weight), euthanize the mice. Dissociate the teratomas in the mice and cut the teratomas into small blocks (the volume of each block shall not exceed 5 × 5 × 2 mm). Fix the teratoma blocks with 4% paraformaldehyde at 4°C overnight.

D.3.4 Paraffin sectioning and HE staining

Embed the fixed samples in paraffin and section the paraffin‐embedded tissue blocks into slices of 4‐10 μm in thickness. Float the slices onto clean glass slides. Perform haematoxylin and eosin (HE). Record the staining results by photography under a microscope.

**D.4 Analysis of results**

Teratomas containing tissues and cells from all three embryonic germ layers (eg, glandular epithelial tissue from the endoderm, cartilage tissues from the mesoderm, and neural tissues from the ectoderm) indicate that the human embryonic stem cells possess pluripotency.
