# Corrigendum

**DOI:** 10.1111/cpr.13010

**Published:** 2021-04-05

In Hao et al,^1^ the following error was published on page 1.

The second affiliation of Professor Qi Zhou should be ‘Chinese Society for Stem Cell Research, Shanghai, China’.

The online version of this article has been corrected.

We apologize for this error.
